# Thyrotoxicosis and the Heart: An Underrecognized Trigger of Acute Coronary Syndromes

**DOI:** 10.3390/biomedicines13112591

**Published:** 2025-10-23

**Authors:** Larisa Anghel, Anca Diaconu, Laura-Cătălina Benchea, Cristina Prisacariu, Dragoș Viorel Scripcariu, Răzvan-Liviu Zanfirescu, Gavril-Silviu Bîrgoan, Radu Andy Sascău, Cristian Stătescu

**Affiliations:** 1 Internal Medicine Department, Grigore T. Popa University of Medicine and Pharmacy, 700503 Iași, Romania; anca.a.diaconu@gmail.com (A.D.); benchea.laura-catalina@d.umfiasi.ro (L.-C.B.); cristina.prisacariu@umfiasi.ro (C.P.); silviubirgoan@gmail.com (G.-S.B.); radu.sascau@umfiasi.ro (R.A.S.); cristian.statescu@umfiasi.ro (C.S.); 2 Cardiology Department, Cardiovascular Diseases Institute “Prof. Dr. George I. M. Georgescu”, 700503 Iași, Romania; zanfirescu_razvan-liviu@d.umfiasi.ro; 3 Surgical Sciences Department, Grigore T. Popa University of Medicine and Pharmacy, 700115 Iași, Romania; dscripcariu@gmail.com; 4 Pathophysiology Department, Grigore T. Popa University of Medicine and Pharmacy, 700503 Iași, Romania

**Keywords:** thyrotoxicosis, acute coronary syndrome, coronary vasospasm, Graves’ Disease, Myocardial Infarction with Non-Obstructive Coronary Arteries (MINOCA)

## Abstract

**Background:** Thyrotoxicosis is a systemic condition with well-documented cardiovascular effects, but its role as a precipitant of acute coronary syndromes (ACS) is often overlooked. This review summarizes clinical cases and original studies from the last 20 years, describing ACS triggered by thyrotoxicosis. **Methods:** Following PRISMA 2020 guidelines, we searched PubMed, Scopus, and Embase for reports published between 2004–2025. Only case reports and original articles were included. Data extracted included demographics, ECG findings, angiography results, thyroid function, etiology of hyperthyroidism, and outcomes. **Results:** A total of 35 cases were identified. The mean age was in the fourth decade of life, with a female predominance (57%, 20 out of 35). More than half of the patients presented with ST-segment elevation myocardial infarction (STEMI) or STEMI equivalents (21 out of 35; 60%). Electrocardiographic abnormalities most often involved anterior or inferior leads. Coronary angiography revealed normal vessels or diffuse vasospasm in 18 cases (51%), while thrombotic occlusion was observed in 4 cases (11%), spontaneous dissection in 2 cases (6%), and myocardial bridging in 3 cases (9%). The leading cause of thyrotoxicosis was Graves’ disease (≈65%), followed by painless thyroiditis, iatrogenic causes, and gestational hyperthyroidism. Thyroid storm was reported in approximately 20% of cases and was associated with malignant ventricular arrhythmias or sudden cardiac death. **Conclusions:** Thyrotoxicosis should be recognized as a rare but important trigger of ACS, especially in young patients without traditional risk factors. Pathophysiological mechanisms include coronary vasospasm, increased myocardial oxygen demand, and hypercoagulability. Early recognition may prevent unnecessary revascularization and optimize outcomes through integrated endocrine and cardiac management.

## 1. Introduction

Thyrotoxicosis refers to the clinical condition resulting from excess circulating thyroid hormones, and its effects on the cardiovascular system are both extensive and clinically significant. Among these hormones, triiodothyronine (T3) plays a central role in modulating heart rate, myocardial contractility, vascular tone, and endothelial function. Although thyroxine (T4) is the primary hormone secreted by the thyroid gland, it is converted in peripheral tissues to T3, which then enters cardiomyocytes and binds to nuclear thyroid hormone receptors (TRs), influencing gene expression crucial for normal cardiac performance [1].

In hyperthyroid states—including overt thyrotoxicosis and thyroid storm—these cardiovascular effects are greatly exaggerated. Increased sympathetic activity, enhanced erythropoiesis, vasodilation, and stimulation of the renin–angiotensin–aldosterone system contribute to a hyperdynamic circulatory state. Cardiac output can rise by as much as 300% compared to that in euthyroid individuals. Additionally, T3 promotes nitric oxide synthesis and angiogenesis, which lower systemic vascular resistance but can also precipitate hemodynamic instability and myocardial ischemia, even in the absence of obstructive coronary artery disease [2].

While conditions such as atrial fibrillation and high-output heart failure are well-established complications of thyrotoxicosis, its potential to provoke acute coronary syndromes (ACS) is often underrecognized. Thyrotoxicosis may contribute to plaque rupture, coronary vasospasm, or hypercoagulable states, thereby unmasking subclinical atherosclerosis or mimicking myocardial infarction with non-obstructive coronary arteries (MINOCA). In rare and severe cases, thyroid storm can even result in coronary embolism or direct myocardial injury [1,2,3].

**Figure 1:**
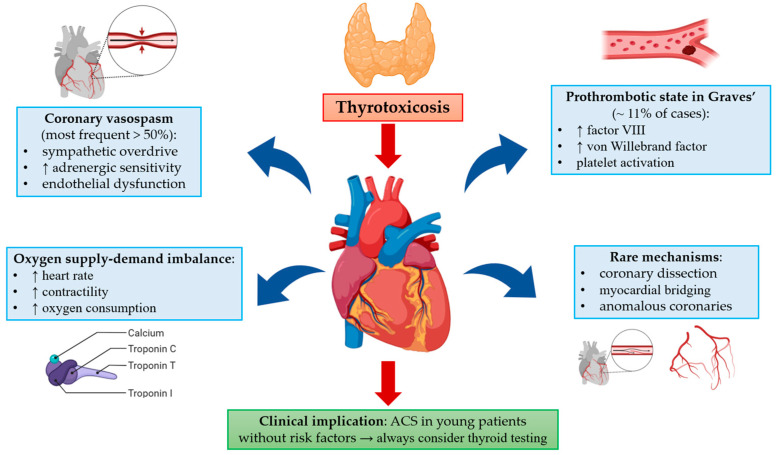
Pathophysiological mechanisms linking thyrotoxicosis with acute coronary syndromes and their clinical implications.

The link between thyrotoxicosis and acute myocardial infarction (AMI) is multifaceted, encompassing a dynamic interaction of hemodynamic strain, vascular reactivity, metabolic demand, and inflammatory signaling [2]. These mechanisms are illustrated in Figure 1, which outlines the primary pathways by which excessive thyroid hormone activity may trigger ACS, particularly in patients lacking traditional cardiovascular risk factors.

Case reports and small observational series indicate that 5–15% of ACS cases may occur in the context of unrecognized thyrotoxicosis, especially among younger individuals. Nevertheless, thyroid function testing is not routinely performed in ACS presentations, potentially delaying appropriate diagnosis and targeted therapy [2].

In response to this knowledge gap, we conducted a review of case reports and original studies published over the last twenty years to better characterize the clinical profile, mechanisms, diagnostic features, and outcomes of ACS triggered by thyrotoxicosis. Our goal is to synthesize the available evidence and raise clinical awareness of this underrecognized, but reversible cause of myocardial infarction.

## 2. Materials and Methods

This review was conducted in accordance with the PRISMA 2020 (Preferred Reporting Items for Systematic Reviews and Meta-Analyses) guidelines. Although the protocol for this review was not prospectively registered, all steps followed the standard methodology for transparent and reproducible evidence synthesis.

A comprehensive literature search was performed using three major electronic databases—PubMed, Scopus, and Embase—to identify relevant studies published in the last two decades (between 1 January 2004, and 30 June 2025). The search strategy included the following key terms and Boolean operators: “thyrotoxicosis” OR “hyperthyroidism” OR “thyroid storm” AND “acute coronary syndrome” OR “STEMI” OR “myocardial infarction” OR “MINOCA” OR “cardiovascular event”. In addition, the reference lists of all included studies were manually screened to identify other eligible articles not captured in the initial database search.

To ensure consistency and relevance, we established strict inclusion and exclusion criteria. Studies were eligible if they were case reports or original clinical investigations (prospective or retrospective) involving patients with biochemically confirmed thyrotoxicosis who experienced an acute coronary syndrome, defined as ST-elevation myocardial infarction (STEMI), non-ST-elevation myocardial infarction (NSTEMI), or myocardial infarction with non-obstructive coronary arteries. We limited our search to articles published in English to ensure accuracy in data extraction and avoid misinterpretation of clinical details, and restricted the timeframe to 2004–2025 to capture contemporary case management and reflect current diagnostic and therapeutic standards. Studies were excluded if they were review articles, editorials, or letters without original patient data, or if they involved cases of iatrogenic thyrotoxicosis without ACS or isolated arrhythmia or heart failure in the absence of ischemic findings. Animal studies were also excluded.

Two independent reviewers screened the titles, abstracts, and full texts of all identified records to determine eligibility. Discrepancies between reviewers were resolved by consensus. For each included article, we extracted detailed information into a structured database. This included the name of the first author, year of publication, country of origin, patient age and sex, type and etiology of thyrotoxicosis (e.g., Graves’ disease, thyroiditis, or thyroid storm), clinical presentation of ACS (STEMI, NSTEMI, or MINOCA), findings from coronary angiography (including presence or absence of obstructive lesions), thyroid function test results (TSH, free T3, free T4), relevant cardiac biomarkers and imaging, therapeutic interventions (such as beta-blockers, antithyroid medications, corticosteroids, or percutaneous coronary intervention), and patient outcomes (including recovery, complications, and in-hospital or short-term mortality). STEMI was defined by ischemic symptoms with ST-segment elevation and biomarker elevation when available. NSTEMI was defined by ischemic symptoms with non-ST elevation ECG changes and elevated troponin. MINOCA was defined as acute myocardial infarction with elevated troponin and non-obstructive coronary arteries (<50% stenosis) on angiography [2]. Thyrotoxicosis was defined biochemically by suppressed TSH with elevated FT4 and/or FT3, irrespective of clinical presentation [1].

**Figure 2:**
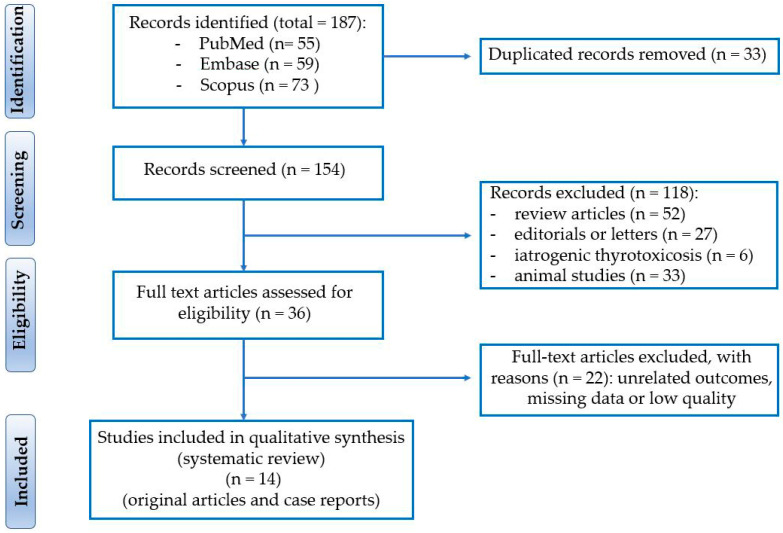
Preferred Reporting Items for Systematic reviews and Meta-Analysis (PRISMA) diagram.

The flow of study selection is illustrated in Figure 2 using an adapted PRISMA diagram, which outlines the number of records identified, screened, assessed for eligibility, and ultimately included in the qualitative synthesis.

## 3. Results

A total of 35 published case reports and original studies met the inclusion criteria. All described patients had thyrotoxicosis, presenting with acute coronary syndromes (ACS), with adequate documentation of clinical, biochemical, and angiographic findings (Table 1).

### 3.1. Demographics

The cohort comprised 20 women (57%) and 15 men (43%), with a mean age of approximately 40 years, indicating that most cases occurred in young to middle-aged adults. A small subset of cases was reported in pregnancy (n = 2).

### 3.2. Clinical Presentation

The most frequent presentation was ST-segment elevation myocardial infarction (STEMI) or STEMI equivalents, identified in 21 cases (60%). Non-ST-elevation ACS or unstable angina accounted for approximately 20% of reports, while a minority presented with isolated arrhythmias or non-ischemic ECG changes (≈10%).

Electrocardiographic abnormalities predominantly involved anterior or inferior leads, with occasional reports of Wellens’ pattern and marked QT prolongation in the context of thyroid storm.

### 3.3. Coronary Angiography Findings

**Figure 3:**
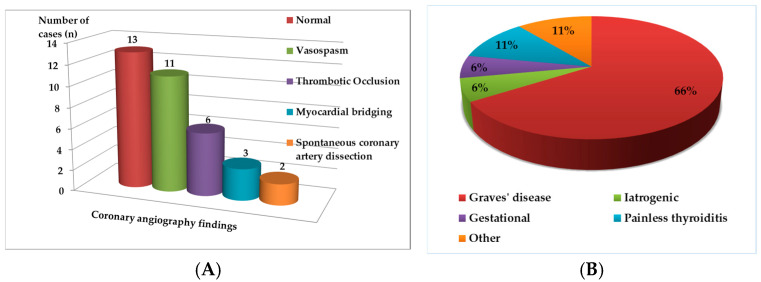
(**A**). Coronary angiography findings. (**B**). Etiology of thyrotoxicosis.

Coronary imaging revealed a heterogeneous spectrum of abnormalities. In 18 patients (51%), angiography demonstrated normal coronary arteries or diffuse vasospasm, suggesting a functional rather than structural mechanism. Thrombotic occlusion was observed in 4 cases (11%), while spontaneous coronary artery dissection was reported in 2 patients (6%). Additionally, myocardial bridging or anomalous coronary anatomy accounted for 3 cases (9%) (Figure 3).

### 3.4. Thyroid Profile and Etiology

All patients demonstrated suppressed TSH levels with variable elevations in free thyroxine (FT4). The underlying etiology of thyrotoxicosis was most commonly Graves’ disease (23 cases; 65%). Other causes included painless thyroiditis (4 cases; 11%), gestational thyrotoxicosis (2 cases; 6%), and amiodarone-induced or iatrogenic hyperthyroidism (2 cases; 6%). Thyroid storm was reported in 7 patients (20%), and was frequently associated with malignant arrhythmias (ventricular tachycardia or fibrillation) and hemodynamic instability. With regard to disease chronology, thyrotoxicosis was newly diagnosed at the time of ACS presentation in 27 of 35 patients (77%), indicating that ACS often represented the first clinical manifestation of thyroid dysfunction. In 8 cases (23%), the thyroid disorder had been previously diagnosed, and patients were under treatment (medical or post-radioiodine) before the ischemic event. Unfortunately, the precise time interval between biochemical thyrotoxicosis (elevated FT4) and the onset of ACS was not specified in most reports (approximately 80%). However, among the subset with available data, ACS occurred within a few days to two weeks of symptom onset, usually during the acute or storm phase of uncontrolled hyperthyroidism, supporting a temporal association between hormonal excess and myocardial ischemia.

### 3.5. Outcomes

The majority of patients achieved recovery following a combination of standard ACS therapy and restoration of euthyroid status using antithyroid drugs, beta-blockers, and supportive care. Invasive revascularization was rarely required except in cases of thrombotic occlusion or dissection. Fatal outcomes were uncommon, but when present, they were primarily linked to thyroid storm or malignant ventricular arrhythmias. Unfortunately, most case reports did not provide precise timelines between the onset of thyrotoxic symptoms and the occurrence of chest pain/ACS. In the subset of reports where a timeline was available, ACS occurred within a few days of progressive thyrotoxic symptoms or in the acute phase of thyroid storm, supporting a temporal association between uncontrolled thyrotoxicosis and ischemic events.

## 4. Discussion

The link between thyrotoxicosis and acute coronary syndromes has been described for more than forty years. The earliest angiographic evidence was published in 1979 by Wei et al., who documented coronary vasospasm in a patient with hyperthyroidism complicated by ventricular fibrillation, with resolution after restoration of euthyroidism [37]. Since then, published literature has mainly consisted of individual case reports, which together illustrate a wide clinical spectrum ranging from STEMI and STEMI equivalents such as Wellens syndrome, to NSTEMI and vasospastic angina.

In 2010, Jaber et al. described a case of vasospastic angina related to severe Graves’ disease and summarized 20 similar cases previously reported [38]. Later, Zheng et al. reviewed 21 cases published between 2002 and 2014, noting that over half of the patients had normal coronary angiography, with vasospasm—rather than thrombotic occlusion—emerging as the predominant mechanism [36]. Despite these observations, no large-scale studies or prospective cohorts are available, and current knowledge remains based almost entirely on isolated case descriptions. The present review builds upon these earlier reports by systematically evaluating 35 cases published between 2007 and 2025, aiming to provide an updated synthesis of clinical presentation, angiographic findings, and underlying thyroid-related mechanisms in ACS.

### 4.1. Pathophysiological Mechanisms

Our review highlights that more than half of the patients with thyrotoxicosis-related ACS presented with vasospastic angina or MINOCA, where coronary arteries appeared normal or showed diffuse spasm. The remaining patients demonstrated obstructive mechanisms—such as thrombosis, dissection, or myocardial bridging—showing that thyrotoxicosis can precipitate ACS through both functional and structural pathways. Thus, thyrotoxicosis should not be viewed as the sole cause of ACS but rather as a precipitating factor, most often linked to vasospastic angina and MINOCA through mechanisms such as sympathetic overactivity, endothelial dysfunction, and hypercoagulability, while true obstructive MI remains less common. Excess thyroid hormone contributes to this process by inducing sympathetic hyperactivity, increased adrenergic receptor sensitivity, and endothelial dysfunction, all of which promote coronary constriction. At the same time, thyroid hormone excess increases myocardial oxygen demand, creating an imbalance between supply and demand that can precipitate ischemia, particularly under stress. In patients with Graves’ disease, an additional factor is the prothrombotic state, characterized by elevated factor VIII and von Willebrand factor, which may explain the occurrence of coronary thrombosis in a subset of patients (≈11%) [39]. Less common mechanisms, such as spontaneous coronary artery dissection and myocardial bridging, have also been reported, highlighting the heterogeneity of pathways through which thyrotoxicosis can precipitate ACS [1,2,39]. These clinical observations complement mechanistic insights from prior reviews on thyroid–cardiac interactions, which emphasized the multifactorial impact of thyroid hormones on vascular tone, hemostasis, and myocardial performance [2,39].

### 4.2. Clinical Implications

The majority of patients identified in this review were young to middle-aged adults without conventional cardiovascular risk factors, a demographic profile consistent with earlier systematic observations [1,2]. This pattern underscores that thyrotoxicosis-related ACS may often be misdiagnosed or underrecognized, particularly when angiography reveals non-obstructive coronary arteries or diffuse vasospasm. In this context, routine thyroid function testing should be considered in patients with unexplained ACS, especially when the clinical scenario does not align with traditional atherosclerotic disease.

Therapeutic strategies must be directed toward both the acute ischemic event and the underlying endocrine disorder. Coronary vasospasm typically demonstrates good responsiveness to nitrates, calcium-channel blockers, and beta-blockers, yet definitive resolution is only achieved after restoration of euthyroidism, whether through antithyroid therapy, radioactive iodine, or surgery. Invasive coronary revascularization is rarely warranted, and should be reserved for patients with angiographically confirmed obstructive lesions, coronary thrombosis, or spontaneous coronary dissection [2,40].

These findings align with the broader concept that thyrotoxicosis functions as a systemic trigger for dynamic coronary pathology rather than fixed structural disease. However, the predominance of case reports and small case series highlights the absence of prospective registries or multicenter data, limiting the generalizability of current knowledge. Future studies are essential to better quantify prevalence, establish standardized diagnostic algorithms, and evaluate long-term outcomes following restoration of euthyroidism.

Several special clinical scenarios warrant particular attention. Pregnancy-associated thyrotoxicosis has been reported in otherwise healthy women and, when recognized early, is usually associated with favorable maternal and fetal outcomes under appropriate medical therapy [4,21]. In contrast, drug-induced thyrotoxicosis, most notably amiodarone-induced destructive thyroiditis, poses unique challenges as it may both precipitate ACS and complicate the management of underlying arrhythmias [25]. A further consideration is painless (silent) thyroiditis, which may occur without overt hyperthyroid symptoms; in such cases, ACS may represent the initial clinical manifestation, underscoring the importance of thyroid function screening in atypical ischemic presentations. Other important thyroid pathologies are acute suppurative thyroiditis (AST) and subacute thyroiditis (SAT), two inflammatory thyroid conditions with distinct pathophysiological and clinical profiles. Although AST is rare—accounting for less than 1% of all thyroid diseases—it carries significant morbidity, particularly in pediatric populations, where up to 21% of cases are associated with congenital anomalies such as pyriform sinus fistulae. In contrast, SAT is more prevalent among adults, especially women aged 30–50 years, and is frequently preceded by viral infections. Clinically, AST presents with high fever, severe localized neck pain, and may progress to abscess formation, necessitating prompt antibiotic therapy and surgical or image-guided drainage in select cases. SAT typically manifests as a transient thyrotoxic phase with neck tenderness and elevated inflammatory markers, but is self-limiting in most cases, with non-steroidal anti-inflammatory drugs (NSAIDs) as first-line treatment. Corticosteroids are reserved for patients with severe or refractory symptoms. This year, Toschetti et al. provided a comprehensive overview of acute suppurative thyroiditis and subacute thyroiditis. Importantly, the authors emphasize the need for early differentiation between AST and SAT, as misdiagnosis can lead to delayed treatment or unnecessary interventions. Their review underscores the value of integrating clinical findings, laboratory markers, thyroid scintigraphy, and imaging to guide management and improve outcomes [41].

### 4.3. Prognosis and Outcomes

Overall, outcomes were favorable in the majority of reported cases. Of the 35 patients included, nearly all achieved clinical recovery after initiation of standard ACS therapy combined with restoration of euthyroidism. Importantly, invasive interventions such as percutaneous coronary intervention (PCI) were rarely required, being reserved for cases with fixed stenosis, thrombotic occlusion (≈11%), or spontaneous dissection (≈6%).

Nevertheless, outcomes varied significantly depending on the presence of thyroid storm, which was documented in 7 cases (20%). This subgroup was consistently associated with adverse events, including ventricular tachycardia, ventricular fibrillation, cardiogenic shock, and sudden cardiac death. In fact, most fatalities and severe complications in the reviewed literature occurred in this context, underlining thyroid storm as a critical prognostic determinant.

These findings suggest that, while thyrotoxicosis-related ACS is generally reversible with timely recognition and endocrine management, the presence of thyroid storm markedly increases the risk of poor outcomes. Consequently, early diagnosis and aggressive treatment of thyrotoxicosis in the ACS setting are essential, as they may directly influence survival [1,39].

### 4.4. Knowledge Gaps and Future Directions

Despite growing recognition, thyrotoxicosis-associated ACS remains both underdiagnosed and underreported. Its prevalence is uncertain, in part because angiographic provocation testing for vasospasm is not routinely performed. Furthermore, the available literature is limited almost entirely to individual case reports, with very few prospective or multicenter studies that could provide stronger evidence regarding prognosis and management.

Future investigations should focus on clarifying the relative importance of vasospasm compared with thrombotic mechanisms, as well as better characterizing the prothrombotic state associated with Graves’ disease, where coagulation abnormalities and endothelial dysfunction may play a critical role. Equally important is the need to define the long-term cardiovascular outcomes of patients after restoration of euthyroidism, including the risks of recurrence, arrhythmia burden, and mortality. Establishing large-scale registries and collaborative studies would be an essential step toward moving from anecdotal recognition to evidence-based strategies for diagnosis, treatment, and prevention.

## 5. Conclusions

This review highlights that thyrotoxicosis is an underrecognized precipitant of acute coronary syndromes, particularly in younger patients without conventional risk factors. While many cases present with ST-segment elevation, coronary angiography often shows normal arteries or diffuse vasospasm rather than fixed obstruction. Graves’ disease is the most common underlying etiology, and outcomes are generally favorable once euthyroidism is restored. Thyrotoxicosis should be regarded as a trigger for the full spectrum of ACS presentations—from vasospastic angina and MINOCA to obstructive coronary syndromes. Early thyroid function testing in patients with ACS and non-obstructive coronaries may enhance diagnostic accuracy, avoid unnecessary interventions, and guide appropriate management. Thyroid storm, however, remains a high-risk scenario, carrying significant mortality from malignant arrhythmias and requiring urgent recognition and treatment.

## Figures and Tables

**Table 1:** Clinical characteristics, coronary findings, and thyroid profiles of reported cases of thyrotoxicosis-associated acute coronary syndromes (2007–2025).

Case	Age (Years), Sex	ECG	Coronary Angiography	History of CV Disease	TSH Level (µIU/mL)	Free T4 Level (pmol/L)	Thyroid Storm	Hyperthyroidism Cause
Ardehali et al., 2025 [3]	36, F, pregnant	ST-segment depression V3 to V6	LM persistent spasm (90% stenosis)	No	Undetectable	48.3	No	Graves’ disease
Wibawa et al., 2025 [4]	30, F, pregnant	ST-segment elevation II, III, aVF, V4 to V6	Normal	No	<0.05	34.9	No	Gestational hyperthyroidism
Naderi et al., 2024 [5]	36, F	Sinus tachycardia	Normal	No	0.005	83.78	No	Graves’ disease
Širvys et al., 2024 [6]	62, F	ST-segment depression I, aVL, V4 to V6, ST-segment elevation aVR	Diffuse vasospasm in all coronary arteries	No	Undetectable	40.19	No	Not mentioned
Omar et al., 2023 [7]	30, M	ST-segment elevation I, aVL, V1 to V5	RCA spasm	No	<0.02	99.5	Yes	Graves’ disease
Anjum et al., 2022 [8]	47, F	Normal	LM spasm	HTN	0.01	32.2	No	Graves’ disease
Patra et al., 2022 [9]	Mid-50s, M	Biphasic T wave inversion in V2, V3 (Wellens)	80% stenosis LAD (PTCA)	No	0.020	1162.3	Yes	Graves’ disease
Muscoli et al., 2022 [10]	45, F	ST-segment elevation anterior leads, VF episodes	Spontaneous LAD dissection	No	0.01	28.2	Yes	Chronic Autoimmune Thyroid Disease
Dixey et al., 2021 [11]	25, F	RBBB, Q waves V1 to V5, VF	LAD thrombotic occlusion, LAD dissection	No	<0.01	490.4	No	Graves’ disease
Ataallah et al., 2020 [12]	47, F	T wave inversion V1, V2, ST-segment depression V4, V5	Severe vasospasm with mild non-obstructive coronary artery disease	No	0.01	71.3	No	Graves
Brown et al., 2020 [13]	23, M	QT prolongation (QTc 652 ms) and minimal ST-segment elevations in V1–V4	Normal CT coronary angiography	No	Undetectable	41.2	Yes	Graves’ disease
Klomp M. et al., 2020 [14]	49, F	ST-segment depression II, III, aVF, V4 to V6	LM and RCA spasm	No	<0.001	59.9	No	Graves’ disease
Krishnan et al., 2019 [15]	31, M	ST-segment elevation V2 to V4	Normal	No	<0.005	66	No	Graves’ disease
Li et al., 2019 [16]	37, M	ST-segment elevation II, III, aVF, V2 to V4 with Q waves II, III, aVF	Normal	No	0.011	0.92	Yes	Graves’ disease
Chang et al., 2017 [17]	48, F	ST-segment elevation V1 to V3, VT	LAD, LCX, RCA spasm	No	0.013	35.3	No	Not mentioned
Menichetti et al., 2017 [18]	58, F	ST-segment elevation II, III, aVF	RCA spasm	2-month history of rest angina	Undetectable	17.0	Yes	Graves’ disease
Rymer de Marchena et al., 2017 [19]	54, M	ST-segment elevation II, III	Normal	No	<0.01	100.5	No	Graves’ disease
Zeitjian et al., 2017 [20]	51, F	Normal	Anomalous RCA origin	No	<0.015	NA	No	Secondary to hypothyroidism treatment
Nannaka et al., 2016 [21]	27, F, pregnant	ST-segment elevation II, III, aVF	RCA spasm		<0.07	318.45		Gestational (hCG induced) hyperthyroidism
Zhou et al., 2015 [22]	66, F	ST-segment elevation II, III, aVF, V2 to V6	Normal	Cerebral infarction	<0.005	>100	No	Not mentioned
Zhang et al., 2014 [23]	26, M	ST-segment elevation II, III, aVF	Myocardial bridging over LAD	No	Not mentioned	Not mentioned	No	Painless thyroiditis
Bouabdallaoui et al., 2013 [24]	23, F	Diffuse ST-segment elevation	Distal LAD thrombosis	No	<0.005	NA	No	Graves’ disease
Brooks et al., 2013 [25]	55, M	LBBB and 2 episodes of VF (ICD interrogation)	Atherosclerotic plaque with minor stenosis and spasm of distal LCX	ICD	<0.01	75.1	No	Amiodarone-induced destructive thyroiditis
Patane et al., 2012 [26]	75, F	Negative T wave II, III, avF, V1 to V6	Normal	AF, HTN	0.003	35.6	No	Graves’ disease
Lee et al., 2012 [27]	48, F	ST-segment elevation V1 to V3	Normal	No	0.031	334.6	No	Graves’ disease
Hama et al., 2012 [28]	25, F	Normal	LAD thrombosis (autopsy)	No	0.03	24.3	No	Subclinical hyperthyroidism
Kim et al., 2011 [29]	35, M	ST-segment elevation II, III, aVF	Normal	No	0.04	58.1	No	Painless thyroiditis
Kuang et al., 2011 [30]	39, F	Widespread ST-segment depression	LM and RCA spasm	No	Undetectable	49.7	No	Not mentioned
Patane et al., 2010 [31]	63, M	AF, negative T waves V4, V5	Normal	No	0.082	15.4	No	Not mentioned
Patane et al., 2009 [32]	28, M	ST-segment elevation II, III, aVF	Myocardial bridging over LAD	No	0.008	NA	No	Iatrogenic hyperthyroidism
Patane et al., 2009 [33]	67, F	New onset AF	Normal	Chest pain	0.009	18.0	No	Subclinical hyperthyroidism
Patel et al., 2008 [34]	40, F	T wave inversion in precordial leads	LM and RCA spasm	No	<0.1	47.6	No	Graves’ disease
Chudleigh et al., 2007 [35]	36, F	Anterior Q waves, lateral ST depression with T wave inversion	LM stenosis	No	<0.02	120	No	Graves’ disease
Chudleigh et al., 2007 [35]	59, F	Inferior ST segment elevation	Normal	No	<0.02	46.2	No	Not mentioned
Zheng et al., 2015 [36]	21, M	ST-segment elevation II, III, aVF, V7 to V9	Normal	No	0.034	434.1	No	Painless thyroiditis

ACS, acute coronary syndrome; AF, atrial fibrillation; CV, cardiovascular; CT, computer tomography; ECG, electrocardiogram; F, female; hCG, human chorionic gonadotropin; HTN, hypertension; ICD, internal cardiac defibrillator; LAD, left anterior descending coronary artery; LBBB, left bundle branch block; LCX, left circumflex artery; LM, left main artery; M, male; MINOCA, myocardial infarction with non-obstructive coronary arteries; NA, not available; RBBB, right bundle branch block; RCA, right coronary artery; PTCA, percutaneous transluminal coronary angioplasty; STEMI, ST-segment elevation myocardial infarction; TSH, thyroid-stimulating hormone; VF, ventricular fibrillation; VT, ventricular tachycardia.

## Data Availability

All data generated or analyzed during this study are included in this article.

## Abbreviations

The following abbreviations are used in this manuscript:

ACS	Acute coronary syndrome
AF	Atrial fibrillation
AMI	Acute myocardial infarction
AST	Acute suppurative thyroiditis
CV	Cardiovascular
ECG	Electrocardiogram
F	Female
FT3	Free triiodothyronine
FT4	Free thyroxine
hCG	Human chorionic gonadotropin
HTN	Hypertension
ICD	Implantable cardioverter-defibrillator
LAD	Left anterior descending coronary artery
LBBB	Left bundle branch block
LCX	Left circumflex coronary artery
LM	Left main coronary artery
M	Male
MINOCA	Myocardial infarction with non-obstructive coronary arteries
NA	Not available/not reported
NSTEMI	Non–ST-segment elevation myocardial infarction
PCI	Percutaneous coronary intervention
PTCA	Percutaneous transluminal coronary angioplasty
RBBB	Right bundle branch block
RCA	Right coronary artery
SAT	Subacute thyroiditis
STEMI	ST-segment elevation myocardial infarction
TRs	Thyroid hormone receptors
TSH	Thyroid-stimulating hormone
